# Fall risk in older adults hospitalized with tumours: Contributing factors and prediction model

**DOI:** 10.1002/nop2.1969

**Published:** 2023-08-16

**Authors:** Xiaoyan Liu, Chen Dong, Rui Zhao, Zhifeng Gu, Chi Sun

**Affiliations:** ^1^ Geriatric Department Affiliated Hospital of Nantong University; Medical School of Nantong University Nantong China; ^2^ Research Center of Clinical Medical Affiliated Hospital of Nantong University Nantong China; ^3^ Department of Rheumatology Affiliated Hospital of Nantong University Nantong China

**Keywords:** cancer, elderly, fall, inpatients, nurse, prediction model

## Abstract

**Aim:**

Rates vary widely across hospitals globally and typically range from 3 to 11 falls per 1000 bed days and as 7–11 in Affiliated Hospital of Nantong University. This study determined to explore contributing factors and poor prognosis of fall in elderly tumour patients in China.

**Design:**

A cross‐sectional study.

**Methods:**

161 older adults were invited to participate in this study and completed a self‐reported questionnaire, took blood tests, and received the exam of musculoskeletal ultrasound.

**Results:**

Among 161 patients, falls occurred in 41 cases, accounting for 24.8%. 51.6% of older adults suffered from intermediate‐to‐high risk of falls. Fall history, reduced self‐care ability, sleep disturbance, hearing impairment, hyperkyphosis, chronic disease, platelet count, and the thickness of left muscle rectus femoris (LF‐MLT), and left cross‐sectional area (LF‐CSA) were all contributing factors of fall, and higher risk of fall indicating lower quality of life. A fall prediction model was established in this study based on above contributing factors with good prediction efficiency (AUC = 0.920).

**Patient or public contribution:**

The patient volunteers participated in this study and provided valuable data for the final analysis and the acquisition of conclusion.

## 1 INTRODUCTION

Falls are defined as an unexpected change in position that is not caused by an inherent event (such as a brain haemorrhage) or substantial hazard, which lead to an individual to land on the ground, floor or a lower level (Montero‐Odasso et al., 2022). Fall rates among older adults in China range from 14.7% to 34.0% with the mean fall rate being 18.0% (Xu et al., 2022). Rates vary widely across hospitals globally and typically range from 3 to 11 falls per 1000 bed days, (Heng et al., 2022) while 7–11 falls per 1000 bed days in Affiliated Hospital of Nantong University. Falls are the second leading cause of unintentional injury and death worldwide, trailing only traffic accidents. (Montero‐Odasso et al., 2021)

Malignant tumours have been attributed to be the leading cause of death for nearly 25% of China's population, (Wu et al., 2019) with a higher incidence in older adults, especially those aged over 65 (Morris & Lewis, 2020).

Falls are a major concern among elderly cancer patients (Burhenn et al., 2020). A study reported that 69% of older adults with cancer who underwent surgery, radiation or chemotherapy experienced at least one fall during hospitalization or at home within 2–3 months, whereas 51.4% of older cancer patients experienced at least two falls (Dotan et al., 2021). It is significantly higher than the reported fall rate (33.3%) for older adults worldwide (Sattar et al., 2021). The consequences of a fall in a cancer patient are not limited to injury, delayed recovery and hospitalization, but also more complications, unpredictable disease progression, more care need and ultimately poor prognosis (Morris et al., 2022).

Studies indicated that only 10% of older adults hospitalized with tumours who fell in the last 6 months received attention, assessment or intervention from healthcare professionals in hospital (Dotan et al., 2021). Therefore, we explored the influencing factors of fall in older adults hospitalized with tumours and constructed a prediction model.

## 2 METHODS

### 2.1 Participants

The current cross‐sectional study enrolled 161 older adults hospitalized with tumours recruited from Affiliated Hospital of Nantong University between May and December 2021. Patients aged ≥60 years, able to communicate, walk independently (or with an auxiliary tool) and receive cancer treatment without palliative or hospice care were invited in this study. The study of nursing excluded older inpatients with undiagnosed tumours, physical disability and missing clinical biochemical index data.

### 2.2 Demographic and clinical characteristics

A questionnaire was used to collect participants' demographic and clinical information, including sex, age, smoking and drinking status, exercise status, tumour category, fall history, upper arm circumference, leg circumference, hyperkyphosis, status of medication and chronic diseases, and so on.

### 2.3 Comprehensive geriatric assessment

The risk of falls in older adults hospitalized with tumours was assessed using the Morse Fall Scale (MFS) with total score ranging from 0 to 125, and higher scores indicate higher risk of falling. Chinese version of MFS, in which low risk of fall (score < 25), moderate risk (score of 25–45) and high risk (score > 45) can be divided, was used in this study (Huang et al., 2021).

The psychological and cognitive state were evaluated using 7‐item generalized anxiety disorder (GAD‐7) scale for anxiety, 15‐item geriatric depression scale (GDS‐15) for depression and Montreal Cognitive Assessment (MoCA) for cognition. Each item in the GAD‐7 is scored from 0 to 3, with the total scores ranging between 0 and 21 (Terlizzi & Villarroel, 2020). The maximum total score of GDS‐15 is 15, and higher scores indicate severer depression (Zhang et al., 2020). The MoCA score ≥26 indicates normal cognitive state and score of ≤25 indicates impairment, while a point is added as a normal demarcation score if the education period is >12 years (Cersonsky et al., 2022). Sleep quality was evaluated by the Pittsburgh Sleep Quality Index (PSQI), the total score of which ranged from 0 to 21 (Orskov & Norup, 2022). The higher the PSQI score, the poorer sleep quality, and PSQI score >5 is indicative of sleep disorder (Zhou et al., 2020).

Physical functional status was evaluated by Barthel Index (BI) for self‐care ability, Short Physical Performance Battery (SPPB) and Timed Up and Go (TUG). Total score of BI ranging from 0 to 100 and higher score indicates better self‐care ability (McGill et al., 2022). The SPPB was used to measure balance, gait, strength and endurance, with a total SPPB score ranging from 0 to 12, and lower score indicating poorer physical function (Lee et al., 2021). The TUG test is used to evaluate functional mobility and physical performance in older people based on the time it takes to rise from a chair, walk 3 metres, and then turn and walk back to sit in the chair (Hendriks et al., 2022). The frailty index was used to assess frailty and was calculated by counting the number of deficits, with higher score indicating more severe status of frailty (Atkins et al., 2021). Nutritional status was assessed by Mini Nutritional Assessment–Short Form (MNA‐SF) and clinical features (total protein and albumin). Lower scores of MNA‐SF indicating poorer nutrition and MNA‐SF <11 was defined as Malnourished (Fashho et al., 2020).

The 36‐Item Short Form Survey (SF‐36) was used to assess quality of life, which consists of 36 items and can be divided into 8 dimensions including physical function (PF), role‐physical (RP), body pain (BP), general health (GH), vitality (VT), social function (SF), role‐emotional (RE) and mental health (MH). Higher scores indicate a better QoL (Wang et al., 2022). The Memorial University of Newfoundland Scale of Happiness (MUNSH) was used to measure the subjective well‐being of older adults. The MUNSH contains 24 items, and total scores range from −24 to +24 points (Zhang et al., 2022). The Life Satisfaction Index‐A (LSIA) was used to measure perceived life satisfaction. The total score of LSIA ranges from 0 to 20, and higher score indicates better life satisfaction (Aydogdu, 2023 #1753).

### 2.4 Clinical features

The patient's blood biochemical and other laboratory indicators are also collected of this study, including white blood cells (WBC), red blood cells (RBC), platelets (PLT) counts and haemoglobin, and cancer marker (carcinoembryonic antigen [CEA]).

### 2.5 Musculoskeletal ultrasound

A GE LOgiQ E9 ultrasound machine (GE Corp) fitted with a 15‐MHz linear array ultrasound probe was used to measure thickness of muscle rectus femoris, cross‐sectional area and thickness of medial femoral muscle rectus femoris on both sides of the patient's thigh. The following parameters were measured for muscle mass: LF‐MLT (the thickness of left muscle rectus femoris), LVI‐MLT (the thickness of the left vastus intermedius muscle), LF‐CSA (left cross‐sectional area), RF‐MLT (the thickness of right muscle rectus femoris), RVI‐MLT (the thickness of the right vastus intermedius muscle) and RF‐CSA (right cross‐sectional area) (Jahanandish et al., 2021).

### 2.6 Statistical analysis

All data were analysed by SPSS 22.0 (IBM Corp). The normality of distribution for continuous data was assessed by the Kolmogorov–Smirnov test. Numerical variables were expressed as mean ± standard deviation or median with interquartile range, while categorical variables were presented as percentage values. The Mann–Whitney *U*‐test, chi‐square test or double‐tailed *t*‐test were used to analyse the statistical data. The prediction model was built after selecting all significant variables (*p* < 0.05) for a stepwise binary logistic regression analysis. ROC curves were used to analyse the sensitivity and specificity of all variables in predicting fall risk and to validate the prediction model. *p* < 0.05 was considered statistically significant.

## 3 RESULTS

### 3.1 Patient characteristics

Table 1 shows the sociodemographic characteristics of the 161 older adults hospitalized with tumours. 51.6% were classified as being at intermediate‐to‐high risk of falls. Falls occurred in 41 cases, accounting for 24.8%, including 12 (15.4%) patients in the low‐risk group and 28 (33.7%) patients in the medium–high‐risk group.

**TABLE 1:** Sociodemographic analysis of falls in elderly cancer inpatients.

Variable	Total sample (*n* = 161)	Low‐risk group (*n* = 78)	Intermediate‐to‐high‐risk group (*n* = 83)	χ^2^ /Z/t	*p*‐value
Sex, male	95 (59)	47 (60.3)	48 (57.8)	0.098	0.440
Age, years	71.29 ± 6.82	69.80 ± 6.35	72.70 ± 6.98	2.756	0.007
Place of residence				2.254	0.324
Urban	47 (29.2)	27 (34.6)	20 (24.1)		
Rural	97 (60.2)	44 (56.4)	53 (63.9)		
Cities and towns	17 (10.6)	7 (9)	10 (12)		
Marital status				2.375	0.123
Married	135 (83.9)	69 (88.5)	66 (79.5)		
Other	26 (16.1)	9 (11.5)	17 (20.5)		
Education level				1.910	0.167
≤9 years	129 (80.1)	59 (75.6)	70 (84.3)		
>9 years	32 (19.9)	19 (24.4)	13 (15.7)		
Employment				0.158	0.924
Farmers	61 (37.9)	30 (38.5)	31 (37.3)		
Workers	54 (33.5)	25 (32.1)	29 (34.9)		
Other	46 (28.6)	23 (29.5)	23 (27.7)		
Smoking, yes	16 (9.9)	9 (11.5)	7 (8.4)	0.433	0.510
Drinking, yes	36 (22.4)	21 (26.9)	15 (18.1)	1.814	0.178
Physical exercise, yes	82 (50.9)	42 (53.8)	40 (48.2)	0.514	0.473
Physical labour, yes	126 (78.3)	57 (73.1)	69 (83.1)	2.390	0.122
Tumour category				2.477	0.480
Digestive neoplasm	92 (57.1)	41 (44.6)	51 (55.4)		
Respiratory neoplasm	47 (29.2)	27 (57.4)	20 (42.6)		
Urinary neoplasm	14 (8.7)	7 (50.0)	7 (50.0)		
Other tumours	8 (5.0)	3 (37.5)	5 (62.5)		
Fall history, yes	40 (24.8)	12 (15.4)	28 (33.7)	7.252	0.007
The number of falls	0 (0,5)	0 (0,0)	0 (0,1)	−2.791	0.005
BMI	23.1 ± 3.77	23.16 ± 3.82	23.05 ± 3.74	0.183	0.855
Mean grip strength	23.6 ± 9.1	25.93 ± 8.82	21.42 ± 8.88	3.231	0.001
WHR	0.93 (0.89,0.97)	0.93 (0.89,0.96)	0.92 (0.89,0.98)	−0.529	0.597
Mean upper arm circumference	26 (24,28.3)	26 (24,28.63)	25 (23.5,28.1)	−1.015	0.310
Mean leg circumference	32 (30,34)	32 (30.4,34)	31 (29,34)	−1.481	0.139
Hyperkyphosis, yes	21(13)	5 (6.4)	73 (93.6)	5.869	0.015
Eyesight, impairment	65 (40.4)	25 (32.1)	65 (40.4)	4.352	0.037
Hearing, impairment	12 (7.5)	2 (2.6)	10 (12)	5.244	0.022
Types of medicine taken	1 (0,2)	0 (0,1)	2 (1,2)	−6.542	<0.001
Chronic diseases, yes	90 (55.9)	22 (28.2)	68 (81.9)	47.077	<0.001

Abbreviations: BMI, body mass index; WHR, waist‐to‐hip ratio.

### 3.2 Differences in sociodemographic and clinical features according to fall risk

Fall risk was associated with age, fall history, fall times, mean grip strength, multiple medications, hyperkyphosis, impaired eyesight, hearing impairment and had other chronic diseases. However, no statistically significant association was found between fall risk and tumour category (Table 1).

### 3.3 Association between fall risk and comprehensive geriatric assessment scores

Fall risk was associated with physical functional status, sleep disturbance, frailty and nutritional status (*p* < 0.05) (Table 2).

**TABLE 2:** Univariate analysis between comprehensive geriatric assessment scores and fall risk.

Variable	Total sample (*n* = 161)	Low‐risk group (*n* = 78)	Intermediate‐to‐high‐risk group (*n* = 83)	χ^2^ /Z	*p*‐value
Psychological and spirit status					
GAD‐7, anxiety, yes	20(12.4)	4 (5.1)	16 (19.3)	7.399	0.007
GDS‐15, depression, yes	27 (16.8)	5 (6.4)	22 (26.5)	11.634	0.001
MoCA, abnormal	118 (73.3)	49 (62.8)	69 (83.1)	8.475	0.004
Physical functional status					
BI, self‐care ability damaged	45 (28)	7 (9)	38 (45.8)	27.054	<0.001
SPPB	10 (8,12)	11.5 (10,12)	9 (5,11)	−5.487	<0.001
TUG	8.83 (6.89,11.87)	8.55 (6.91,10.88)	9.5 (6.67,13.66)	−1.891	<0.001
PSQI, sleep disturbance	37 (23)	11 (14.1)	26 (31.3)	6.739	0.009
FI grade				26.17	<0.001
Frailty	9 (5.6)	0 (0)	9 (10.8)		
Before frail	32 (19.9)	6 (7.7)	26 (31.3)		
Non‐frail	120 (74.5)	72 (92.3)	48 (57.8)		
Nutritional status					
MNA‐SF, malnourished	63 (39.1)	23 (29.5)	40 (48.2)	5.907	0.015
Total protein/g·L^−1^	64.80 ± 6.26	65.94 ± 5.79	63.89 ± 6.56	2.101	0.037
Albumin/g·L^−1^	37.5(34.9,40.2)	38.5(36.0,40.7)	36.4 (33.4,39.4)	−2.069	0.039

Abbreviations: BI, Barthel Index; FI, frailty index; GAD‐7, 7‐item generalized anxiety disorder; GDS‐15, 15‐item geriatric depression scale; MNA‐SF, Mini Nutritional Assessment–Short Form; MoCA, montreal cognitive assessment; PSQI, Pittsburgh Sleep Quality Index; SPPB, the short physical performance battery; TUG, timed up and go.

### 3.4 Associations of fall risk with tumour‐related blood indices and muscle mass

Older adults hospitalized with tumours with an intermediate‐to‐high risk of fall had lower RBC counts, lower haemoglobin levels, higher PLT count and lower muscle mass than those with low risk of fall (*p* < 0.05; Table 3).

**TABLE 3:** Univariate analysis of fall risk and tumour‐related blood indices, muscle mass.

Variable	Low‐risk group (*n* = 78)	Intermediate‐to‐high‐risk group (*n* = 83)	Z/t	*p*‐value
Blood indices				
WBC/10^9^ · L^−1^	5.6 (4.4,7.02)	6 (4.7,6.9)	−0.695	0.487
RBC/10^12^ · L^−1^	4.10 ± 0.49	3.86 ± 0.62	2.777	0.006
Haemoglobin/g · L^−1^	125 (117,138)	117 (107,131)	−3.353	0.001
PLT/10^9^ · L^−1^	178 (135.8225.5)	206 (169,267)	−3.019	0.003
CEA/ng	2.2 (1.5,4.1)	2.7 (1.7,4.8)	−1.341	0.180
Muscle mass				
RF‐MLT/mm	8.75 (7.5,10.78)	7.8 (6.2,9.2)	−2.289	0.022
RVI‐MLT/mm	8.75 (7.5,10.78)	7.5 (6,9.6)	−3.221	0.001
RF‐CSA/cm^2^	2.6 (1.7,3.7)	2.2 (1.6,3.1)	−1.838	0.066
LF‐MLT/mm	8.85 (7,10.5)	7.7 (5.9,9.3)	−2.952	0.003
LVI‐MLT/mm	8.8 (7.7,10.6)	7.4 (5.7,8.6)	−4.300	<0.001
LF‐CSA/cm^2^	2.5 (1.8,3.5)	2.1 (1.4,3.1)	−2.348	0.019

Abbreviations: CEA, carcinoembryonic antigen; LF‐CSA, left cross‐sectional area; LF‐MLT, left muscle rectus femoris; PLT, platelets; RBC, red blood cells; WBC, white blood cells.

### 3.5 Independent risk factors for falls.

We handled each dimension score as the dependent variable and baseline factors impacting fall risk as independent variables, using binary logistic regression analysis. The statistically significant factors are presented in Table 4.

**TABLE 4:** Analysis of logistic regression models of fall risk.

Risk factors	Beta	SE	*p*‐value	Exp(B)	95%CI
Fall history	−2.447	0.75	0.001	0.087	0.020,0.376
BI, impaired	3.065	0.79	<0.001	21.427	4.559,100.709
PSQI, sleep disturbance	1.787	0.731	0.015	5.973	1.425,25.047
Hearing impairment	4.040	1.367	0.003	56.838	3.898,828.874
Hyperkyphosis	−2.553	1.006	0.011	0.078	0.011,0.559
Chronic diseases	−4.296	0.770	<0.001	0.014	0.003,0.062
PLT/10^9^ · L^−1^	0.012	0.004	0.007	1.012	1.003,1.021
LF‐MLT (mm)	−0.006	0.003	0.017	0.994	0.989,0.999
LF‐CSA (cm^2^)	−0.537	0.206	0.009	0.585	0.391,0.875
Constant	4.319	3.099	0.163	75.107	

Abbreviations: BI, Barthel Index; LF‐CSA, left cross‐sectional area; LF‐MLT, left muscle rectus femoris; PLT, platelets; PSQI, Pittsburgh Sleep Quality Index.

### 3.6 Fall‐risk prediction model in older patients with tumour

According to the ROC curve analysis, high PLT count (area under the curve [AUC] = 0.638, *p* = 0.003), sleep disturbance ([AUC] = 0.630, *p* = 0.004), low BI score ([AUC] = 0.684, *p* < 0.001), fall history (AUC = 0.592, *p* = 0.045), having other chronic diseases (AUC = 0.769, *p* < 0.001), LF‐MLT (thinner, AUC = 0.635, *p* = 0.003) and LF‐CSA (smaller, AUC = 0.607, *p* = 0.019) were predictors of high fall risk. Although hyperkyphosis (AUC = 0.564, *p* = 0.159) and hearing impairment (AUC = 0.547, *p* = 0.299) influenced falls in older adults hospitalized with tumours, these factors did not demonstrate any statistically predicting value of this study.

Based on the result of logistic analysis, we explored all significant seven variables including PLT, PSQI, BI, LF‐MLT, LF‐CSA, fall history and chronic disease into one prediction moder of fall risk of older patients with tumour. The fall‐risk prediction model for elderly cancer inpatients was further obtained using the prediction model formula: *p* = 1/{1 + exp[3.065 × BI‐2.447 × fall history+1.787 × PSQI+0.012 × platelet count‐4.296 × chronic disease‐0.006 × LF‐MLT‐0.537 × LF‐CSA + 4.319]}, and AUC = 0.920, 95%CI = 0.876–0.964, sensitivity of 0.872 and specificity of 0.880.

### 3.7 Association of fall risk with QoL, happiness and life satisfaction

Fall risk of older patients with tumour showed significant side effects on patients' QoL, happiness and life satisfaction (Table 5).

**TABLE 5:** Association of fall risk with QoL, happiness and life satisfaction.

Variable	Total sample (*n* = 161)	Low‐risk group (*n* = 78)	Intermediate‐to‐high‐risk group (*n* = 83)	Z	*p*‐value
SF‐36					
PCS	73 (44.5,84.25)	79.88 (66.44,85.5)	56 (32.5,78)	−4.809	<0.001
MCS	82.5 (54.13,94)	90.5 (74.3,94.3)	70.9 (40,89.75)	−4.854	<0.001
PF	65 (40,82.5)	67.5 (53.75,85)	50 (25,80)	−3.694	<0.001
RP	75 (0,100)	100 (18.75,100)	0 (0,100)	−4.104	<0.001
BP	100 (62,100)	100 (83,100)	100 (52,100)	−3.133	0.002
GH	65 (50,72)	72 (65,77)	65 (40,72)	−4.732	<0.001
VT	75 (55,85)	80 (70,90)	65 (45,80)	−5.071	<0.001
SF	87.5 (50,100)	100 (75,100)	62.5 (37.5100)	−4.705	<0.001
RE	100 (0,100)	100 (100,100)	100 (0,100)	−2.883	0.004
MH	88 (72,92)	92 (83,92)	80 (64,92)	−4.862	<0.001
HT	25 (25,37.5)	25 (25,50)	25 (25,25)	−3.725	<0.001
MUNSH	45 (39,47)	46 (42,47)	44 (34,46)	−2.266	0.023
LSIA	16 (13,17)	16.5 (14,18)	15 (12,17)	−3.142	0.002

*Note*: PCS (the physical component summary) = (PF+ RP+ BP+ GH)/4; MCS (the mental component summary) = (VT+ SF+ RE+ MH) /4.

Abbreviations: BP, body pain, GH, general health; LSIA, Life Satisfaction Index‐A; MCS, the mental component summary; MH, mental health; MUNSH, the Memorial University of Newfoundland Scale of Happiness; PCS, the physical component summary; PF, physical function; RE, role‐emotional; RP, role‐physical; SF, social function; VT, vitality.

## 4 DISCUSSION

This nursing study, which was conducted at an institution in eastern China, revealed that 51.6% of older adults hospitalized with tumours were at an intermediate‐to‐high risk of falls and 24.8% had fall history. We also confirmed that fall risk was evenly distributed across both sexes. Fall history, hyperkyphosis, hearing impairment, chronic diseases, impaired self‐care ability, sleep disturbance, higher PLT count, and lower LF‐MLT and LF‐CSA were independent predictors of fall risk in older adults hospitalized with tumours. In addition, older adults hospitalized with tumours with an intermediate‐to‐high risk of falls had poorer QoL, happiness and life satisfaction than those with low risk of falls.

In addition, hyperkyphosis increases the likelihood of additional fall‐risk factors while lowering their ability to prevent falls, increasing overall fall risk (Koele et al., 2022). Hearing impairment can impair gait control during daily activities, increase gait variability and increase the risk of accidental falls (Criter & Gustavson, 2020). Kenis et al., 2022 confirmed that history of falls could independently predict falls in the future and the risk of repeated falls was higher in patients with a fall history. Immonen et al., (2020) showed that chronic diseases have side an impact on falls and that fall rates are positively correlated with chronic diseases of Chinese. Alenazi et al., (2023) identified the association between falls and chronic diseases, including hypertension and neuropathy. The above data are consistent with the result in this study.

Older patients with cancer and impaired self‐care ability were reported at a higher risk of falls (Goineau et al., 2018). It is probably because older cancer patients are more prone to frailty, which impacts their self‐care ability. Poor sleep quality, resulting in insufficient nighttime sleep duration and daytime dysfunctions, was also important risk factor for falls in older adults (Sattar et al., 2021) (Lee, Chung, & Kim, 2021). Increasing sleep duration, while reducing daytime dysfunctions and sleep disturbances, could mitigate unintentional falls (S. Lee et al., 2021). In this study, self‐care ability and sleep disturbance also were revealed to be associated with fall risk among.

This study demonstrated that the muscle mass, including thickness and cross‐sectional area of the lower limb muscle, was significantly associated with fall risk. Sai et al., (2021) showed that the thickness of quadriceps femoris muscle was significantly correlated with fall injuries, with optimal cut‐off values of 3.37 cm and 3.54 cm for men and women, respectively. In our study, the average muscle thickness of older cancer patients from China was approximately 7–9 mm, which is lower than the 3–4 cm reported for older people in Europe and the United States. Based on these findings, it is speculated that the muscle of older tumour patients has been adversely affected by the disease and then faced more risk of fall.

According to the result in this study, higher fall risk resulted in lower QoL, lesser happiness and poor life satisfaction, which is consistent with the report of Silva et al., (2021). The feeling of lonely and unaccompanied made them more prone to falls. Therefore, it is necessary to improve the social connections of older adults within family, hospital and communities, preventing social isolation, ultimately reducing the risk of falls.

## 5 LIMITATIONS

This is the first cross‐sectional study that explores fall risk in older adults hospitalized with tumours in China. However, this study still showed some limitations. First, the study had a relatively small sample size. Second, this study lacked data on cancer stages. This was because the stage of tumour was unknown for patients who had not received surgical or other interventions after being diagnosed with tumour.

## AUTHOR CONTRIBUTIONS

Xiaoyan Liu and Zhifeng Gu contributed to the conception and the design of the research; Xiaoyan Liu and Chen Dong carried out the research design, interview and data analysis; Xiaoyan Liu completed the manuscript writing; Chi Sun made important contributions to the research design and manuscript writing; Rui Zhao made contributions to the research design. Xiaoyan Liu and Chi Sun transcribed the data.

## FUNDING INFORMATION

Nantong Municipal Science and Technology Plan Guiding Project MSZ20019; National Natural Science Foundation of China, grant number 82101891, 82071838; Jiangsu Province Senile Health Scientific Research Project, grant number LR2021035; Jiangsu Provincial Medical Key Discipline, grant number JSDW202205.

## CONFLICT OF INTEREST STATEMENT

The authors declare no conflict of interest.

## ETHICS STATEMENT

This study was performed in line with the principles of the Declaration of Helsinki. Approval was granted by the Ethics Committee of the Affiliated Hospital of Nantong University (No. 2021‐K058‐01).

## Data Availability

The data that support the findings of this study are available upon request by contact with corresponding author.
